# Clinical Characteristics of Hospitalized COVID-19 Patients Who Received at Least One Dose of COVID-19 Vaccine

**DOI:** 10.3390/vaccines9070781

**Published:** 2021-07-13

**Authors:** Piotr Rzymski, Monika Pazgan-Simon, Krzysztof Simon, Tadeusz Łapiński, Dorota Zarębska-Michaluk, Barbara Szczepańska, Michał Chojnicki, Iwona Mozer-Lisewska, Robert Flisiak

**Affiliations:** 1 Department of Environmental Medicine, Poznan University of Medical Sciences, 60-806 Poznań, Poland; 2 Integrated Science Association (ISA), Universal Scientific Education and Research Network (USERN), 60-806 Poznań, Poland; 3 1st Infectious Diseases Ward, Gromkowski Regional Specialist Hospital, 50-149 Wroclaw, Poland; monikapazgansimon@gmail.com; 4 Department of Infectious Diseases and Hepatology, Wrocław Medical University, 51-149 Wrocław, Poland; krzysimon@gmail.com; 5 Department of Infectious Diseases and Hepatology, Medical University of Bialystok, 15-540 Białystok, Poland; twlapinski@gmail.com; 6 Department of Infectious Diseases, Jan Kochanowski University, 25-369 Kielce, Poland; dorota.zarebska-michaluk@ujk.edu.pl; 7 Department of the Children’s Diseases, The Infectious-Neurological Subdivision, Jan Kochanowski University, 25-369 Kielce, Poland; barbara.szczepanska@ujk.edu.pl; 8 Department of Immunobiology, Poznan University of Medical Sciences, 60-806 Poznań, Poland; michalc@ump.edu.pl; 9 Department of Infectious Diseases, Jozef Strus Hospital, 61-285 Poznań, Poland; iwonalisewska@ump.edu.pl

**Keywords:** vaccination, breakthrough infection, severe COVID-19, SARS-CoV-2

## Abstract

The clinical trials of the COVID-19 vaccines that are authorized in the European Union have revealed high efficacy in preventing symptomatic infections. However, during vaccination campaigns, some vaccine recipients, including those partially and fully vaccinated, will experience severe COVID-19, requiring hospitalization. This may particularly concern patients with a diminished immune response to the vaccine, as well as non-responders. This work has retrospectively analyzed the 92 cases of patients who were hospitalized between 27 December 2020 and 31 May 2021 in four Polish healthcare units due to COVID-19, and who have previously received the COVID-19 vaccine (54.3% ≤ 14 days after the first dose, 26.1% > 14 days after the first dose, 7.6% ≤ 14 days after the second dose, and 12% > 14 days after the second dose). These patients represented a minute fraction (1.2%) of all the COVID-19 patients who were hospitalized during the same period in the same healthcare institutions. No significant differences in white blood count, absolute lymphocyte count nadir, C-reactive protein, interleukin-6, procalcitonin, oxygen saturation, lung involvement, and fever frequency were found between the recipients of the first and second vaccine dose. A total of 15 deaths were noted (1.1% of all fatal COVID-19 cases in the considered period and healthcare units), including six in patients who received the second dose (five > 14 days after the second dose)—three of these subjects were using immunosuppressive medicines, and two were confirmed to be vaccine non-responders. The study reassures that severe COVID-19 and deaths are not common in vaccinated individuals, highlights that the clinical course in such patients may not reveal any distinctive features, and advocates for close monitoring of those at a higher risk of vaccine failure.

## 1. Introduction

The start of the rollout of COVID-19 vaccines at the turn of 2020–2021 brought new hope to the fight against the pandemic [1,2]. The vaccines that are authorized in the European Union have been proven to have a good safety profile, be immunogenic, and provide a high efficacy against symptomatic SARS-CoV-2 infection [3,4,5,6]. The COVID-19 vaccination is, however, met with several challenges, encompassing logistics, dosage shortages, vaccine hesitancy, media coverage, and the emergence of novel SARS-CoV-2 variants [1,7,8,9,10].

Three out of four vaccines that are authorized in the European Union require two doses, separated by specific intervals outlined in the summary of product characteristics (SmPC): BNT162b2 (BioNTech/Pfizer) by 21 days, mRNA-1273 (Moderna) by 28 days, and AZD1222 (Oxford/AstraZeneca) by 4–12 weeks. According to the clinical data, the partial protection against symptomatic infection begins around two-to-three weeks after the first dose, when the anti-spike IgG antibodies emerge. However, receiving the second dose of these vaccines is necessary and pivotal to ensuring optimal protection against COVID-19 [11,12,13]. Since the vaccine administration is conducted during the high spread of SARS-CoV-2 in the population, the risk of infections among vaccinated individuals, especially those who received only one dose, must be considered.

Additionally, reduced immunogenicity after a single dose and after the full vaccination regime must be taken into account in the case of some elderly subjects, cancer patients, and other immunocompromised individuals [14,15,16]. Importantly, some of these vaccine recipients may not produce any antibodies and may remain highly susceptible to the SARS-CoV-2 infection [17]. The exact percentage of individuals falling into the non-responders category is unknown. At the same time, administration of the first dose of the vaccine may lead to false conviction of full protection, and subsequent belittling of precaution and safety measures. Although most SARS-CoV-2 infections in partially or fully vaccinated individuals will be mild, and likely accompanied by decreased viral load and replication, some subjects may still experience severe disease, requiring hospitalization.

This study aimed to analyze cases of COVID-19 hospitalization of partially and fully vaccinated individuals in selected hospitals in Poland. The demographic, clinical and laboratory characteristics, and the course of disease, were considered. These observations add to real-world data on severe cases of post-vaccination SARS-CoV-2 infections, breakthrough infections in individuals more than 14 days after completing all recommended COVID-19 vaccine doses, and associated deaths.

## 2. Material and Methods

### 2.1. Data Extraction

The clinical data on patients who received at least one dose of the COVID-19 vaccine, later hospitalized due to COVID-19 between 27 December 2020 (the first day of the COVID-19 vaccine rollout in Poland) and 31 May 2021 in four Polish healthcare units in Wrocław, Białystok, Kielce and Poznań were extracted and retrospectively analyzed. During the same period, 7552 COVID-19 patients were hospitalized in these medical units, among which 1413 were fatal (18.7%), while the number of newly diagnosed SARS-CoV-2 infections in Poland rose by 1,614,484. The percentage of individuals who received at least a single dose at the end of January 2021 was 2.6%, February—5.7%, March—11.8%, April—23.6%, and reached 36.1% (13,707,350 individuals) at the end of May 2021. The share of fully vaccinated subjects reached 18.5% in the period considered in the study.

The study only included patients who reported the first COVID-19 symptoms following the vaccination and were discharged from the hospital as convalescent or died. The investigated subjects were divided into the following four subgroups: (1) recipients of the first dose with symptoms onset within 14 days since vaccination, (2) recipients of the first dose with symptoms onset >14 days after vaccination, (3) recipients of two doses with symptoms onset within 14 days from the second vaccine dose, (4) recipients of two doses with symptoms onset >14 days after the second vaccine dose (i.e., breakthrough infections). The first subgroup (1) cannot be unequivocally treated as infected after vaccination if one considers that the incubation time of SARS-CoV-2 can extend to 2 weeks [18].

Upon admission, the body temperature, oxygen saturation (SpO_2_) and lung involvement based on computed tomography (CT) imaging were recorded. If available, laboratory data analysis included white blood cell (WBC), C-reactive protein (CRP), interleukin-6 (IL-6), procalcitonin (PCT) at admission. Absolute lymphocyte count (ALC) nadir during the hospital stay was also considered. Patients were defined as vaccine non-responders if they received at least one dose of vaccine >14 days before the onset of COVID-19 symptoms and had a negative result of the serological test (anti-SARS-CoV-2 spike protein IgG antibodies) at admission. The clinical improvement was assessed with the ordinal scale based on WHO recommendations, but modified to an 8-score version to fit the specificity of the national health care system and previously used in the SARSTer study [19]. The scores, given at baseline and after 7, 14, 21 and 28 days after hospitalization, were defined as follows: (1) not hospitalized, no activity restrictions; (2) not hospitalized, no activity restrictions and/or requiring oxygen supplementation at home; (3) hospitalized, does not require oxygen supplementation and does not require medical care; (4) hospitalized, requiring no oxygen supplementation, but requiring medical care; (5) hospitalized, requiring normal oxygen supplementation; (6) hospitalized, on non-invasive ventilation with high-flow oxygen equipment; (7) hospitalized, for invasive mechanical ventilation or extracorporeal membrane oxygenation; (8) death. Patients were diagnosed and treated according to the most recent national recommendations for the management of COVID-19 [20].

Due to the retrospective nature of the presented study, written consent by participants was not necessary.

### 2.2. Statistical Analysis

The statistical analysis was performed using Statistica v.13.1 (StatSoft Inc., Tulsa, OK, USA). Because the disease severity was reported in the ordinal scale, while other variables did not meet the assumption of Gaussian distribution (Shapiro–Wilk test; *p* < 0.05), non-parametric methods were employed. The differences between the sub-groups of patients were analyzed with the Kruskal–Wallis ANOVA with the post hoc Dunn’s test. Differences in data frequencies reported as nominal categorical variables were evaluated by running multiple Pearson’s χ^2^ tests between each sub-group of patients. A *p*-value < 0.05 was considered statistically significant.

## 3. Results

### 3.1. Demographic Characteristics

A total of 92 patients were considered in this analysis—they constituted 1.22% of all COVID-19 patients who were hospitalized during the same period (27 December 2020–31 May 2021) in the considered healthcare units. Their demographic characteristics are summarized in Table 1. The majority had received only a first vaccine dose (80.4%). The maximum number of days from the second dose to symptoms onset in the studied group was 90. The age of the studied patients was 32–93 years; the subjects aged <70 years constituted 66.5%. The most-frequent chronic comorbidities included cardiovascular diseases (82.6% of subjects), particularly hypertension (63%) and type 2 diabetes (31.5%). Hospitalized individuals who received a second dose more than 14 days before the onset of the symptoms were significantly older than those who displayed symptoms within two weeks from receiving the first dose—no individuals <50 years old were observed. Most of the patients had increased BMI, and no difference, in this regard, was noted between the considered sub-groups. Overall, seven patients were immunosuppressed, while four met the criteria of non-responders. For other patients, the status of anti-SARS-CoV-2 spike protein IgG antibodies was unknown, or the results of the test were positive (although it does not necessarily imply the good responsiveness to the vaccine, as the antibody levels were determined after hospitalization and could also be, at least partially, due to viral infection). None of the patients who were considered in the analysis underwent the documented SARS-CoV-2 infection prior to vaccination.

### 3.2. Clinical Characteristics

The clinical and laboratory data of each sub-group considered in the study are summarized in Table 2. The disease severity at baseline did not differ between these groups, similarly to other clinical data and laboratory parameters. However, the patients who were hospitalized >14 days after the second dose tended to have the highest median IL-6 and CRP levels, and the lowest frequency of fever >38 °C.

**Figure 1:**
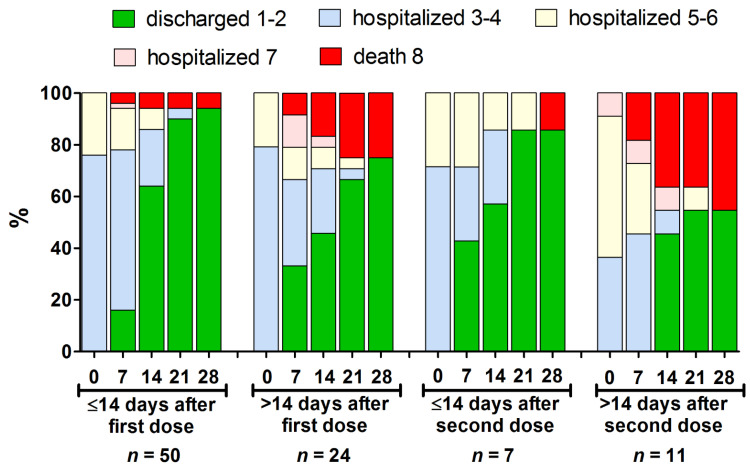
The clinical course presented using an eight-score disease severity scale [19] from baseline to day 28 of hospitalization in the different subsets of hospitalized patients who received at least one vaccine dose (*n* = 92). Further, 1–2 indicates not hospitalized patients; 3–4—hospitalized requiring no oxygen supplementation; 5–6—hospitalized, requiring normal oxygen supplementation or non-invasive ventilation; 7—hospitalized, requiring invasive mechanical ventilation or extracorporeal membrane oxygenation; 8—death.

The clinical course of the disease, from the first to the 28th day of hospitalization, is presented in Figure 1. Overall, 15 cases (16.3%) were fatal—they constituted 1.06% of all the deaths to COVID-19 patients that were recorded during the same period in the considered healthcare units (Table 3). Among those, six occurred in the group that received a one vaccine dose more than 14 days before the onset of the symptoms, one case was noted in the group with symptoms onset ≤14 days after the second dose, and five in the group with symptoms onset >14 following the second dose (Table 2). There was one fatal case of a non-responder (female, aged 80, normal BMI, no immunosuppression) among the recipients of the first dose with symptoms onset >14 days after vaccination. Among those who received both the vaccine doses (regardless of the time prior to the symptoms onset), three fatal subjects were using immunosuppressive medicines; one was a kidney transplant recipient (male, aged 55, normal BMI), and the second suffered from chronic lymphocytic leukemia (male, aged 74, normal BMI), and both had no detectable levels of anti-S IgG antibodies upon admission. The third patient had rheumatoid arthritis (male, aged 83, normal BMI), but his seroconversion status after vaccination was not established. The detailed characteristics of fatal COVID-19 patients in each sub-group are presented in Table 3. Three out of four (75%) confirmed vaccine non-responders in the entire group considered in the present study died to COVID-19. The fatal COVID-19 patients that had previously received two vaccine doses at least 14 days before the onset of the symptoms, revealed the highest disease severity at the baseline, all had a fever > 38 °C, and none had SpO_2_ < 90% (Table 4).

## 4. Discussion

The present paper documents the clinical characteristics of Polish patients who were hospitalized due to COVID-19, who received at least a single dose of the vaccine. The data extracted from selected hospitals demonstrate that the highest number of more-severe infections after vaccination can occur after the first dose, particularly within the first two weeks. During this period, most the vaccinated subjects will not reach a threshold of the immune response that would ensure partial protection, which begins around two-to-three weeks after first dose, when the anti-spike IgG antibodies emerge [11,12,13]. Therefore, they need to be considered as fully vulnerable to contracting the virus. Moreover, the median incubation time of SARS-CoV-2 is 4–5 days, but it can extend through two weeks [18,21]. Therefore, it cannot be excluded that the number of infections with COVID-19 symptoms onset within 14 days from the first vaccine dose, is due to exposure to SARS-CoV-2 prior to vaccination. Nevertheless, the vaccinated patients who received at least one dose of the vaccine constituted a minute fraction of all the subjects who were hospitalized due to COVID-19, in the considered four healthcare institutions during the same period, i.e., 0.98 and 0.24% in the case of the first and second dose recipients, respectively. During the same period, the share of partially and fully vaccinated individuals in the Polish population reached 36.1 and 18.5%, respectively. This further reassures the high efficacy of the COVID-19 vaccines, in preventing severe disease, and, in line with large real-world observations, indicates that, to date, severe breakthrough infections (i.e., identified >14 days after receipt of all recommended doses) are sporadic [22,23].

Most of the investigated subjects in the present study were >60 years old, male, had increased BMI, and suffered from comorbidities. This is consistent with the risk factors for severe COVID-19 [24,25]. However, the patients hospitalized more than 14 days after receiving the second dose tended to be older. Moreover, all the subjects requiring hospitalization after receiving two doses were at least 52 years old, while in the group of single-dose receivers, individuals aged 30–50 were also present. Moreover, the majority of fatal cases occurred in subjects ≥70 years. Experiences with previous vaccines demonstrate that, in elderly subjects, the immune response to vaccination is often less potent, which is the phenomenon attributed to adaptive immunosenescence [26]. This has already been observed in small investigations that were conducted after authorization of the COVID-19 vaccines, e.g., one study reported that more than 30% of vaccinated subjects aged >80 years did not attain neutralizing antibody responses [14].

The present study did not find any distinct clinical or laboratory features for sub-groups that were divided on the basis of the vaccination status of patients (number of doses, days after the last dose). This implies that individuals who suffer from severe COVID-19, despite partial or complete vaccination, will likely undergo a similar clinical course of the disease, and should likely be subject to indifferent treatment. However, one should note that the present study did not compare a clinical course of vaccinated and unvaccinated subjects.

Important observations of this retrospective study include the cases of patients using immunosuppressive medicines. As suggested by different authorities (e.g., UK Joint Committee on Vaccination and Immunisation), individuals with immunosuppression should be prioritized for the COVID-19 vaccination [27]. However, on the other hand, these individuals may reveal a diminished response to the vaccine, while some, as confirmed for two cases reported in our study, could be subject to vaccine failure [17]. Therefore, it is pivotal to advise these patients to continue to adhere to preventive measures, particularly at a time of a high number of infections in the population, and to test the serum level of anti-SARS-CoV-2 IgG antibodies following the vaccination. This is particularly important if one considers that fully vaccinated individuals may be subject to an increasing number of various benefits, including lifting the face mask and physical distancing recommendations. Otherwise, some of these individuals may falsely assume the protection from the adaptive immunity and put themselves in high-risk situations. Notably, in the present study, three out of four (75%) confirmed vaccine non-responders died due to COVID-19.

It should also be noted that most of the breakthrough infections that are reported in the present study cannot be explained by immunosuppression or vaccine failure. Whether this is due to the emergence and spread of selected SARS-CoV-2 variants of concern (VOC), requires further studies [28,29]. As shown using the neutralization assays employing the sera of vaccinated individuals and pseudotyped virus, some variants, e.g., those possessing the E484K mutation in the receptor-binding domain, can potentially evade, to some extent, the immunity provided by the vaccines [30]. A recent study in Washington State indicated that VOC are overrepresented among post-vaccination breakthrough infections that do not require hospitalization [31]. As shown, mutations present in VOC, such as B.1.1.7 and B.1.351, do not escape the T-cell-mediated immunity that is elicited through vaccination [32]. Therefore, it is urgently needed to not rely solely on the antibody status level, but also to assess the cellular responses in vaccinated patients with severe breakthrough infections.

The study limitations must be stressed. Firstly, this is a retrospective analysis from four hospitals in Poland, and cannot draw a general conclusion on the frequency of hospitalizations and deaths due to COVID-19 among vaccinated Poles. However, the present study demonstrates a trend according to which SARS-CoV-2 infections among vaccinated individuals requiring hospitalization are expected more often after the first dose, particularly within 14 days, during which the vaccinated subjects remain fully vulnerable to the virus and may also contract it before vaccine administration. Secondly, no conclusions should be drawn on the differences in the frequency of COVID-19 hospitalizations after a particular vaccine, as the rate of their administration in Poland was related to the availability, and was also different among various occupational and age groups. Moreover, as recommended by the Polish Ministry of Health since 8 March 2021, a second dose of the AZD1222 vaccine was given after 12 weeks, resulting in a lower share of individuals being fully vaccinated with this vaccine as compared to BNT162b2. Thirdly, the considered group of patients was relatively small, and the demographic, clinical, and laboratory characteristics require further confirmations on larger cohorts. Although the data on immunosuppressed individuals are of interest, one should bear in mind that these patients constituted only 7.6% of the studied group. More specific research on immunocompromised subjects is needed in order to understand their risk of vaccine failure. Moreover, the present study included patients identified as non-responders, based on the negative result of the anti-SARS-CoV-2 spike protein IgG antibodies status. However, this does not imply that every other patient should unambiguously be considered as a vaccine responder, since the antibody status was assessed after hospital admission, not enabling the vaccine- and infection-induced adaptive immune response to be distinguished. Last, but not least, the variants that caused infections in the studied individuals were not identified. According to the genomic surveillance of SARS-CoV-2 in Poland, infections with the B.1.1.7 variant were increasingly dominant during the studied period, from approx. 15% at the beginning of 2021, to over 90% in May 2021 [33]. Although the COVID-19 vaccines used in Poland retain high efficacy against this variant, the immunoprotection after the first dose appears to be decreased [34,35]. Other variants of concern, such as B.1.351 that has a more significant effect on vaccine efficacy [36,37], were only sporadically identified [33]. Nevertheless, one should note that the data presented in this manuscript may not apply to all SARS-CoV-2 variants, including B.1.617.2, which was first detected in Poland in May 2021.

## 5. Conclusions

The study indicates that, for the considered period and healthcare institutions, COVID-19 patients who received at least one dose of the COVID-19 vaccine before hospitalization, constituted a minute fraction of all COVID-19 patients, and were represented mainly by those who received only one dose ≤14 days before first symptoms onset. Therefore, this indirectly reassures the high efficacy of vaccines in preventing severe disease. As shown, the demographic and clinical characteristics of hospitalized subjects that are partially or fully vaccinated, may not vary significantly. The fatal patients were mostly >70-year-old men, and had increased BMI and comorbidities, which were mostly cardiovascular diseases. The study findings also suggest that fully vaccinated individuals with immunosuppression should first be screened for their adaptive response, to avoid the potential risks of severe infection in the case of vaccine failure. Further studies are warranted in order to elucidate the more severe cases of breakthrough infections in the vaccine-responding individuals.

## Figures and Tables

**Table 1:** The demographic breakdown of COVID-19 patients who have previously received at least one dose of COVID-19 vaccine (*n* = 92). Different letters in each row indicate statistically significant differences between the sub-groups (*p* < 0.05) demonstrated with the post hoc Dunn’s test following the Kruskal–Wallis ANOVA or with the Pearson’s χ^2^ test.

Parameter	All (*n* = 92)	≤14 Days after 1st Dose (*n* = 50)	>14 Days after 1st Dose (*n* = 24)	≤14 Days after 2nd Dose (*n* = 7)	>14 Days after 2nd Dose (*n* = 11)
**Age** Mean ± SD, years Range, years >70 years, *n* (%)	68.3 ± 11.9 32–93 40 (43.5)	65.5 ± 11.5 a 32–89 16 (32.0)	69.9 ± 10.2 ab 46–89 12 (50.0)	73.4 ± 6.9 ab 67–85 4 (57.1)	78.5 ± 14.3 b 52–93 8 (72.7)
**Gender** Male, *n* (%) Female, *n* (%)	58 (63.0) 34 (37.0)	a 31 (62.0) 19 (38.0)	a 15 (62.5) 9 (37.5)	a 4 (57.1) 3 (42.9)	a 8 (72.7) 3 (27.3)
**BMI** Mean ± SD, kg/m^2^ Underweight Normal (18.5–24.9), *n* (%) Overweight (25.0–29.9), *n* (%) Obesity (≥30.0), *n* (%)	28.7 ± 5.4 1 (1.1) 23 (25.0) 36 (39.1) 32 (34.8)	28.6 ± 4.5 a 1 (2.0) 8 (16.0) 21 (42.0) 20 (40.0)	29.5 ± 7.3 a 0 (0.0) 8 (33.3) 8(33.3) 8 (33.3)	28.7 ± 3.8 a 0 (0.0) 2 (28.6) 3 (42.9) 2 (28.6)	27.4 ± 6.3 a 0 (0.0) 5 (45.5) 4 (36.4) 2 (18.2)
**Chronic comorbidities**, *n* (%)	82 (89.1)	41 (82.0) a	23 (95.8) a	7 (100.0) a	11 (100.0) a
**Immunosuppression**, *n* (%)	7 (7.6)	0 (0.0)	3 (12.5)	1 (14.3)	3 (27.3)
**Vaccine** BNT162b2, *n* (%) mRNA-1273, *n* (%) AZD1222, *n* (%)	51 (55.4) 13 (14.1) 28 (30.4)	a 22 (44.0) 9 (18.0) 19 (38.0)	a 12 (50.0) 3 (12.5) 9 (37.5)	b 6 (85.7) 1 (14.3) 0 (0.0)	b 11 (100.0) 0 (0,0) 0 (0.0)
**Confirmed vaccine non-responders**, *n* (%)	4 (4.4)	-	2 (8.3)	1 (14.3)	1 (9.1)

**Table 2:** The clinical characteristics of COVID-19 patients who previously received at least one dose of COVID-19 vaccine (*n* = 92). Different letters in each row indicate statistically significant differences between the sub-groups (*p* < 0.05) demonstrated with the post hoc Dunn’s test following the Kruskal–Wallis ANOVA or with the Pearson’s χ^2^ test.

Parameter	Group			
	≤14 Days after 1st Dose (*n* = 50)	>14 Days after 1st Dose (*n* = 24)	≤14 Days after 2nd Dose (*n* = 7)	>14 Days after 2nd Dose (*n* = 11)
**Baseline WHO**, median (IQR)	4 (4-4) a	4 (4-4) a	4 (4-5) a	5 (4-6) a
**Fever > 38 °C**, %	62.1 a	62.5 a	57.1 a	36.4 a
**SpO_2_ < 90%**, %	30.0 a	54.2 a	28.6 a	54.5 a
**Lung involvement**, %	30 (20–40) a	30 (25–47.5) a	30 (10–50) a	24 (15–70) a
WBC * (×10^3^/µL), median (IQR)	6.2 (5.1–8.2) a	7.2 (5.1–9.3) a	9.3 (5.7–12.9) a	7.1 (5.4–13.3) a
ALC nadir * (×10^3^/µL, median (IQR)	1.0 (0.8–1.3) a	0.9 (0.6–2.0) a	1.3 (0.9–2.5) a	0.7 (0.6–1.0) a
**IL-6** (pg/mL), median (IQR)	63.3 (37.1–139.1) a	47.8 (23.6–138.1) a	35.6 (17.4–72.6) a	112.5 (73.0–208.7) a
**CRP** (mg/L), median (IQR)	62.8 (39.1–143.9) a	76.8 (45.1–200.5) a	82.0 (3.2–108.0) a	113.0 (73.0–208.7) a
**PCT** (ng/mL), median (IQR)	0.09 (0.05–0.14) a	0.11 (0.05–0.31) a	0.11 (0.05–0.16) a	0.09 (0.05–0.18) a
**Fatal outcome**, *n* (%)	3 (6.0) a	6 (25) b	1 (14.3) ab	5 (45.5) b

IQR—interquartile range; WBC—white blood count; ALC—absolute lymphocyte count; IL-6—interleukin-6; CRP—C-reactive protein; PCT—procalcitonin; *—the data for patients suffering from chronic lymphocytic leukemia (*n* = 2) were excluded from these statistics.

**Table 3:** The share of patients who received at least one dose of COVID-19 vaccine and were later hospitalized and fatal due to COVID-19 between 27 December 2020 and 31 May 2021 in the Polish healthcare units considered in the present study.

Patients	Group				All
	≤14 Days after 1st Dose	>14 Days after 1st Dose	≤14 Days after 2nd Dose	>14 Days after 2nd Dose	
**Hospitalized**	50	24	7	11	92
**% of all hospitalized** **(*n* = 7552)**	0.66	0.32	0.09	0.15	1.22
**Fatal cases**	3	6	1	5	15
**% of all fatal cases** **(*n* = 1413)**	0.21	0.42	0.07	0.35	1.06

**Table 4:** The clinical and laboratory characteristics of patients with fatal COVID-19 who have received at least one dose of COVID-19 vaccine.

Parameter	Group			
	≤14 Days after 1st Dose (*n* = 3)	>14 Days after 1st Dose (*n* = 6)	≤14 Days after 2nd Dose (*n* = 1)	>14 Days after 2nd Dose (*n* = 5)
**Age**, mean ± SD, years >70 years, *n* (%)	71.7 ± 3.2 2 (66.6)	77.0 ± 8.7 5 (83.3)	74 1 (100)	77.4 ± 14.3 4 (80.0)
**Gender** Male, *n* (%) / Female, *n* (%)	1 (33.3)/2 (66.6)	3 (50.0)/3 (50.0)	1 (100.0)/0 (0.0)	4 (80.0)/1 (20.0)
BMI, mean ± SD, kg/m^2^ Normal (18.5–24.9), *n* (%) Overweight (25.0–29.9), *n* (%) Obesity (≥30.0), *n* (%)	31.7 ± 3.2 0 (0) 1 (33.3) 2 (66.6)	24.7 ± 3.4 4 (66.6) 2 (33.3) 0 (0)	21.7 1 (100) 0 (0) 0 (0)	24.5 ± 0.75 3 (60.0) 2 (40.0) 0 (0)
**Chronic comorbidities**, *n* (%) CVD, *n* (%) Diabetes, *n* (%)	100 (100) 100 (100) 100 (100)	5 (83.3) 4 (66.6) 1 (16.6)	100 (100) 0 (0) 0 (0)	100 (100) 4 (80) 3 (60)
**Immunosuppression**, *n* (%)	0 (0)	0 (0)	1 (100)	2 (40)
**Confirmed vaccine** **non-responders**, *n* (%)	-	1 (16.7)	1 (100)	1 (20.0)
**Baseline WHO**, median (IQR)	4 (4-4)	4 (4-5)	3	6 (5-6)
**Fever >38 °C**, %	2 (66.6)	3 (50.0)	1 (100.0)	0 (0.0)
**SpO_2_ <90%**, %	2 (66.6)	4 (66.6)	0 (0)	5 (100.0)
**Lung involvement**, %	40 (30–40)	55 (40–60)	60	70 (50–70)
**WBC** (×10^3^/µL),median (IQR)	6.0 (5.9–11.9)	7.3 (3.5–15.4)	19.5 *	8.8 (7.1–14.5)
**ALC nadir** (×10^3^/µL), median (IQR)	1 (0.4–1.4)	0.6 (0.3–2.0)	15.0 *	0.6 (0.5–0.7)
**IL-6** (pg/mL),median (IQR)	n.a.	138 (134–142)	40.8	122 (103–228)
**CRP** (mg/L), median (IQR)	207 (61–226)	136 (43–238)	82	125 (22–199)
**PCT** (ng/mL), median (IQR)	0.3 (0.06–0.6)	0.1 (0.1–0.6)	0.12	0.2 (0.2–0.2)

CVD—cardiovascular diseases; * patient suffering from chronic lymphocytic leukemia; n.a.—data not available.

## Data Availability

The data presented in this study are available from the corresponding authors on reasonable request.
